# The Effects of Olive Oil Consumption on Biochemical Parameters and Body Mass Index of People with Nonalcoholic Fatty Liver Disease: A Systematic Review and Meta-Analysis of Randomized Controlled Trials

**DOI:** 10.3390/nu16060857

**Published:** 2024-03-15

**Authors:** Georgios Tsamos, Georgios Kalopitas, Kleo Evripidou, Dimitra Vasdeki, Theocharis Koufakis, Vasileios Kanavas, Christina Antza, Georgios Germanidis, Michail Chourdakis

**Affiliations:** 1 Department of Gastroenterology, Norfolk and Norwich University Hospital NHS Trust, Norwich NR4 7UY, UK; 2 Division of Gastroenterology and Hepatology, 1st Department of Internal Medicine, AHEPA University Hospital, School of Medicine, Faculty of Health Sciences, Aristotle University of Thessaloniki, 546 36 Thessaloniki, Greece; giwrgoskalopitas@gmail.com (G.K.); georgiosgermanidis@gmail.com (G.G.); 3 Laboratory of Hygiene, Social and Preventive Medicine and Medical Statistics, School of Medicine, Faculty of Health Sciences, Aristotle University of Thessaloniki, 541 24 Thessaloniki, Greece; kleoevripidou@gmail.com (K.E.); mhourd@gapps.auth.gr (M.C.); 4 Division of Endocrinology and Metabolism and Diabetes Center, 1st Department of Internal Medicine, Medical School, Aristotle University of Thessaloniki, AHEPA University Hospital, 546 36 Thessaloniki, Greece; demivs14@gmail.com; 5 2nd Propedeutic Department of Internal Medicine, Aristotle University of Thessaloniki, Hippokration General Hospital, 546 42 Thessaloniki, Greece; thkoyfak@hotmail.com; 6 Laboratory of Biomathematics, School of Medicine, University of Thessaly, 412 22 Larissa, Greece; vasiliskanavas@gmail.com; 7 3rd Department of Internal Medicine, Medical School, Aristotle University of Thessaloniki, Papageorgiou Hospital, 564 03 Thessaloniki, Greece; kris-antza@hotmail.com

**Keywords:** nonalcoholic fatty liver disease, olive oil, meta-analysis, Mediterranean diet

## Abstract

Nonalcoholic fatty liver disease (NAFLD), the most common chronic liver disorder, is closely associated with insulin resistance, obesity, and metabolic syndromes. A body of research has proposed that olive oil, a basic component of the Mediterranean diet with antioxidant and anti-inflammatory properties, may alleviate metabolic disturbances and retard the progression of NAFLD. We conducted a systematic review and meta-analysis to assess the effectiveness of olive oil intake in people with NAFLD. We systematically searched the major electronic databases (PubMed/MEDLINE, Scopus, Cochrane Central Register of Controlled Trials), as well as grey literature sources, to identify randomized controlled trials (RCTs) investigating the effects of olive oil consumption on biochemical and anthropometric parameters of individuals with NAFLD. The quality of the studies was evaluated using the risk-of-bias tool 2.0 (RoB 2). The mean difference (MD) and the 95% confidence interval (CI) were calculated using fixed-effects and random-effects models. Seven RCTs involving 515 subjects were included in the analysis. In the random-effects model, no statistically significant differences were identified with respect to alanine transaminase (MD = −1.83 IU/L, 95% CI: −5.85, 2.19 IU/L, *p* = 0.37, *I*^2^ = 69%) and aspartate transaminase (MD = −1.65 IU/L, 95% CI: −4.48, 1.17 IU/L, *p* = 0.25, *I*^2^ = 72%) levels or waist circumference values (MD = −0.23 cm, 95% CI: −1.23, 0.76 cm, *p* = 0.65, *I*^2^ = 0%). However, a significant effect on body mass index was observed (MD = −0.57 kg/m^2^, 95% CI: −1.08, −0.06 kg/m^2^, *p* = 0.03, *I*^2^ = 51%) for subjects who received olive oil compared to those who received an alternative diet or placebo. The findings of the present meta-analysis suggest a modestly positive impact of olive oil intake on body weight in people with NAFLD.

## 1. Introduction

During the past few decades, the prevalence of nonalcoholic fatty liver disease (NAFLD) has increased, becoming a significant global public health concern, affecting approximately a quarter of the global population (20–30% in Europe and 46% in the United States) [1,2]. First recognized in 1980, this condition stands as a significant instigator of chronic liver ailments, marked by the accumulation of fat within the liver, independent of excessive alcohol intake. Fatty liver disease, alternatively referred to as hepatic steatosis (HS) or fatty liver, denotes the disproportionate buildup of triglycerides within hepatic cells (hepatocytes), encompassing a diverse clinical spectrum extending from HS to nonalcoholic steatohepatitis (NASH), cirrhosis, and hepatocellular carcinoma (HCC) [3,4,5]. Advanced age, oxidative stress, and inflammation, along with risk factors such as insulin resistance, central obesity, hyperlipidemia, and hypertension, which are considered a hepatic manifestation of metabolic syndrome (MetS), play a crucial role in the development and progression of NAFLD [6,7,8]. Consequently, the prevention and treatment of NAFLD are highly relevant for public health.

The adoption of the Mediterranean diet (MD), in which olive oil has a prominent position, has been correlated with numerous health benefits, particularly concerning MetS, diabetes mellitus, obesity, and cardiovascular health, among others [9]. The American Food and Drug Administration (FDA) recommends a daily intake of 20g of olive oil as a preventive measure against cardiovascular disease [10]. Currently, there is no pharmacological treatment available for NAFLD, which has led international guidelines to emphasize lifestyle modifications as the primary approach to the treatment of the condition [11]. This approach involves implementing diet changes, participating in physical exercise activities, and considering insulin sensitizers as part of the treatment regimen, with the primary goal being weight loss [12,13]. In this context, the MD has emerged as an important strategy for the prevention and management of NAFLD due to its antioxidant and anti-inflammatory properties [14,15]. 

Studies conducted in humans have revealed an inverse correlation between intrahepatic levels of specific components found in extra virgin olive oil (EVOO), such as sitosterol, and the degree of steatosis and lobular inflammation, as documented by liver biopsy [16]. The hepatoprotective components of EVOO include tocopherols, phytosterols, phenolic compounds, chlorophyllic compounds, and carotenoid pigments. The beneficial effect of EVOO against NAFLD could be explained by mitochondrial protection, which prevents the decrease in nitrated fatty acid levels caused by a high-fat diet. This protective ability is directly related to the phenolic content [17,18]. Olive oil is believed to independently reduce fat accumulation in the liver, possibly by improving the fatty acid oxidation process [19]. Beyond its ability to decrease low-density lipoprotein (LDL) oxidation, it is suggested that the beneficial effects of olive oil on NAFLD also involve reducing NF-κB activation and improving insulin resistance [20,21,22]. Additionally, experimental studies have indicated that various components of the MD possess the capacity to improve NAFLD by influencing the transcription of genes involved in metabolic processes. For instance, Pipitone et al. recently conducted a study highlighting the correlation between the consumption of red and golden tomatoes and the amelioration of NAFLD parameters. Their findings revealed a significant reduction in triglycerides, low-density lipoprotein-cholesterol, and fasting glucose levels, accompanied by the reversal of steatosis. Furthermore, an increase in high-density lipoprotein cholesterol was observed, indicative of an overall improvement in the lipid profile. Notably, the study also shed light on the impact of tomato consumption on hepatic gene expression. Specifically, upregulation of genes such as Gk and Hnf4α, associated with metabolic homeostasis, as well as Lepr, implicated in adipokine signaling, and Il6 and Tnf, involved in inflammatory responses, was noted. These findings suggest that incorporating tomatoes into one’s diet as part of a nutraceutical approach holds potential in both the prevention and therapeutic management of NAFLD [23]. 

Despite promising findings, available studies in the field often report contradictory results. Furthermore, although extensive research has been conducted on the relationship between diet and NAFLD, data on the specific association between olive oil and NAFLD-related parameters remain limited. For these reasons, we conducted a systematic review and meta-analysis to offer a precise estimate of the impact of olive oil consumption on the biochemical and anthropometric parameters of people with NAFLD.

## 2. Materials and Methods

This systematic review was conducted according to the Cochrane, the Centre for Reviews and Dissemination, and the PRISMA guidelines [24,25,26]. The protocol has been registered with the Open Science Framework accessed on 14 March 2022 (https://osf.io/rqpt3/).

The primary objective of this study was to evaluate the association between olive oil consumption and NAFLD and its effect on liver enzymes, specifically alanine transaminase (ALT). Furthermore, secondary analyses were performed on other biochemical or anthropometric variables when provided by the included studies.

### 2.1. Eligibility Criteria 

Only complete reports examining the effects of olive oil intake on surrogate markers of NAFLD were considered. Studies were considered eligible for inclusion in the meta-analysis if they met all of the following criteria: (a) involving participants over 18 years of age; (b) randomized controlled trials (RCTs); (c) involving subjects diagnosed with NAFLD by imaging techniques (i.e., liver ultrasonography, magnetic resonance imaging, computed tomography) or liver biopsy; (d) reported daily alcohol intake of less than 30 g/day for men and less than 20 g/day for women; and (e) interventions comparing olive oil intake with other types of fat and/or specific diets. No specific limitations were imposed on the sex, medical history, race, or geographic distribution of the individuals included in the study.

The following exclusion criteria were applied: (a) involving patients with a history of alcohol abuse; (b) including individuals with steatosis caused by other factors or with pre-existing chronic liver diseases such as hepatitis B and/or C, autoimmune hepatitis, Wilson’s disease, and others; (c) including pregnant women; (d) including patients receiving hepatotoxic medications; (e) involving patients who initiated or changed their antidiabetic or antilipidemic medication within the last 6 months or those who began vitamin E therapy for NAFLD within the last 6 months; (f) studies that did not involve regular intake of olive oil, such as single-dose interventions; and (g) articles written in languages other than English.

### 2.2. Search Strategy and Study Selection

The following electronic databases were systematically searched: Pubmed/MEDLINE, Scopus, and Cochrane Central Register of Controlled Trials (CENTRAL) in the Cochrane Library to identify pertinent studies that met the inclusion criteria. Additionally, a search was conducted on ClinicalTrials.gov to gather more evidence. The review process focused on articles published from inception until the end of 2023. The search strategy combined keywords from the following research question: “Administration of olive oil to patients with NAFLD”. Appropriate free-text words and medical subject headings (MESH) were created using all possible synonyms and associated phrases. The grey literature, including relevant theses, was searched to ensure comprehensive coverage. In addition, ProQuest and Scopus electronic databases were consulted, and experts in the field were contacted. Additionally, relevant conference papers and proceedings were evaluated. These references were manually searched. Finally, a PROSPERO search was performed to avoid duplicates. A detailed search strategy for PUBMED is described in Table 1.

### 2.3. Article Selection and Data Extraction

Two reviewers, T.G. and E.K., independently assessed potentially relevant articles for eligibility. Studies identified from the search in electronic medical databases were imported into the reference manager software (EndNote X7.0). The initial determination of inclusion or exclusion was based on the title and abstract of the study. In the event of discrepancies, all members of the research team engaged in discussions to reach a consensus. The full-text articles were then obtained and evaluated to determine inclusion, with the reasons for exclusion recorded. In cases where the articles referred to additional study reports or published protocols, these additional materials were acquired to ensure complete data extraction. Online screening software Rayyan (https://www.rayyan.ai/) was used to assist in the screening process. Any disagreement between the two reviewers was resolved with the help of a senior third reviewer. When necessary, the authors of the studies were contacted for supplementary data. We conducted searches in LILACS, DANS EASY Archive, dissertations/theses, and reports for the grey literature. The entire search process was presented in a PRISMA flow chart, providing a clear visual representation.

The information extracted encompassed various aspects, including study design (country, recruitment methods, type and methods of analysis, completion rates), details of the intervention and comparator, and characteristics of the participants (population and setting, inclusion/exclusion criteria, baseline characteristics). In addition, outcome assessment data were recorded. All this information was entered into Microsoft^®^ Excel 2019 for further analysis and organization. A narrative synthesis of the findings was compiled and presented below using descriptive statistics such as percentages and summary tables.

### 2.4. Assessment of Quality

Two authors (T.G. and E.K.) conducted separate evaluations of bias susceptibility. Discordances were resolved through the intervention of a third reviewer (C.M.). The evaluation of the risk of bias in each study was performed using the Cochrane Collaboration Risk of Bias tool 2.0 (RoB 2) [33,34]. 

### 2.5. Statistical Analysis, Meta-Analysis, and Meta-Regression

The meta-analysis was performed using the RevMan software (Review Manager, Version 5.4, Cochrane Collaboration, 2020). Mean and standard deviation (SD) data for post-intervention values were inputted. Independent and more refined tests were conducted using the R studio package R 4.0.2 when necessary. Data synthesis involved calculating effect sizes with 95% confidence intervals (CIs) using a random-effects model with inverse variance weighting. Standard deviation with a 95% CI was summarized for continuous data, while relative risk (RR) with a 95% CI was used for dichotomous data. The results of body mass index (BMI), aspartate aminotransferase (AST), and ALT were summarized as differences in means. Heterogeneity among studies was assessed using the χ^2^ statistic (expressed as a *p* value) and the *I*^2^ statistic (expressed as a percentage), with statistical significance set at *p* ≤ 0.05. Studies identified as having a “High risk of bias” and “Some concerns” were excluded, and the heterogeneity was recalculated. Forest plots were generated to visually present the outcomes, with studies arranged based on effect size. Publication bias was evaluated using funnel plots, Begg’s, and Egger’s regression tests. In instances where evidence of publication bias was detected, an additional analysis known as the Duval and Tweedie nonparametric ‘trim and fill’ analysis was conducted. This approach addressed publication bias by estimating missing studies’ numbers and potential outcomes and making necessary adjustments. The ‘trim and fill’ analysis helped formalize the use of the funnel plot, enhancing the reliability of the findings. 

Data presented outside the primary text or tables of the original publications were extracted from the figures. The results reported as medians were converted to means and standard deviations (SDs) using the method outlined by Hozo et al. [35]. When data were unavailable, efforts were made to obtain the missing information by directly contacting the authors. The per-protocol analysis was utilized if obtaining data was not feasible. Any necessary transformations and analyses were performed in accordance with the guidelines outlined in the Cochrane Handbook for Systematic Reviews of Interventions [36]. These adjustments were made to address issues such as missing data or other essential calculations. Sensitivity analyses were conducted on the primary meta-analysis models to examine the robustness of the results. This involved excluding clinical trials deemed to have a serious, critical, or high risk of bias [34,37]. 

## 3. Results

**Figure 1:**
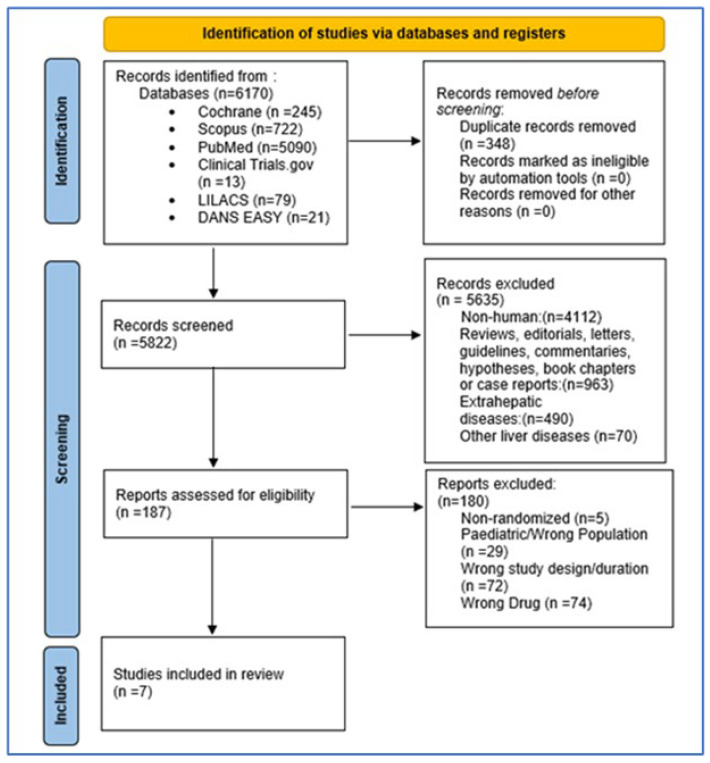
Preferred Reporting Items for Systematic Reviews and Meta-Analyses (PRISMA) flow diagram.

From the initial search of the databases, 6170 studies were identified. After removing duplicate studies, 5822 were screened at the “title and abstract level” and 187 studies at the ‘full-text level’. Finally, seven studies were included in the meta-analysis. The results of the screening process are described in Figure 1. 

### 3.1. Study Characteristics

The study characteristics are presented in Table 2. The total patient population consisted of 529 individuals, of whom 322 (60.9%) were men and 207 (39.1%) were women. Two studies included only males [27,28]. The mean age of the participants was 45 years, and the mean BMI was 32.45 kg/m^2^ at baseline. The mean duration of the dietary intervention with olive oil or other control diet was 23.4 weeks, and the median was 12 weeks (range 8–72 weeks). Study populations consisted mainly of individuals affected by abdominal obesity/dyslipidemia or characteristics of MetS. These trials were carried out in countries of varying economic status, encompassing nations in Africa [9,28,32] as well as Eastern and Western countries [27,29,30,31].

### 3.2. Design of Eligible Studies and Outcome Measures

The study design encompassed a total of seven RCTs and all used a parallel group design [9,27,28,29,30,31,32]. The primary outcome of the evaluated studies was the effect of the assigned dietary intervention on ALT levels. Secondary outcomes included changes in liver enzymes other than ALT (AST, gamma-glutamyl transferase (gGT), lipid and glycemic parameters such as total cholesterol (TC), high-density lipoprotein (HDL-C), LDL-C, fasting plasma glucose (FPG), and fasting plasma insulin (FPI)) and changes in anthropometric characteristics, including waist circumference (WC) and BMI. The liver fat content was measured using noninvasive techniques such as magnetic resonance spectroscopy (MRS) or MRS–proton density fat fraction (MRS-PDFF) [27,29,30,31], ultrasound [9,28,32], and scoring systems that included the fatty liver index (FLI) and the liver fibrosis score [29,31].

### 3.3. Intervention and Comparison Arms

Dietary interventions were delivered at variable time intervals, primarily through one-on-one consultations involving nutritional counseling and personalized approaches. The included studies applied a diverse range of olive oil administration using olive oil in gel capsules or liquid form. Calorie restrictions were implemented in some studies, while two studies [9,28] recommended regular physical activity, and three studies emphasized maintaining a consistent level of physical activity [30,31,32]. However, no studies reported the use of specific behavior change strategies. The predominant comparator treatment used in the clinical trials included alternative oils such as rapeseed, sunflower, and soybean oil [9,27,28]. In the EPA + DHA trials, olive oil served as the comparative intervention [29,31]. In the remaining trials, the comparison group was a normal-fat diet or a low-fat diet (LFD) [30,32].

Among the seven trials, one investigated an intervention based on the traditional Cretan diet according to descriptive food data and analysis of the actual foods consumed combined with national diet guidelines [30], three investigated a hypocaloric diet with daily energy intake recommendations of 20% protein, 50–60% carbohydrates, and 20–30% fat [9,28,32], one examined a normocaloric diet as recommended by the American Association for the Study of Liver Diseases (AASLD) [31], and the remaining two studies evaluated the existing dietary habits of participants without any intervention [27,29]. A study investigated a hypocaloric diet that required minimizing the consumption of fish and nuts rich in omega-3 fatty acids [9], and two allocated 20 g or 20% of total fat (30% of total energy intake) to olive oil each day [9,32]. 

### 3.4. Assessment of Risk of Bias and Publication Bias

**Figure 2:**
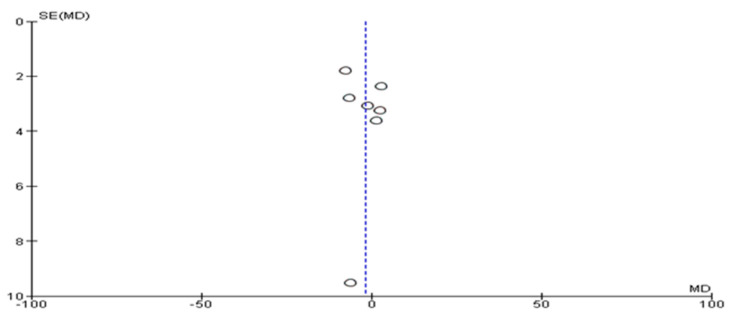
Funnel plot of the included studies regarding ALT concentrations.

The risk of bias was assessed for the primary outcome considered in this review, which was the change in ALT concentrations. Interestingly, only one study was identified as having a high risk of bias. To examine possible publication bias, we generated funnel plots and observed a relatively balanced distribution of results. This finding suggests minimal publication bias since both positive and negative effects were equally represented (Figure 2). 

The study by Shidfar et al. was described as single-blind but without information on deviations from the intended intervention or the appropriate analysis used to estimate the effect of intervention assignment (ITT/PPA) [31]. The studies by Kruse et al. and Nigam et al. were designed as open-label studies, raising concerns about their risk of bias [27,28]. Table 1 provides a comprehensive overview of the ALT outcome reported by each study, as defined by the Cochrane RoB2 tool.

### 3.5. Effects of Olive Oil on Primary and Secondary Outcomes

Table 3 presents a comprehensive overview of the impact of dietary interventions on both primary and secondary outcomes. The MD exhibited superior effects to the LFD on ALT levels and HS as evaluated by imaging techniques (HMRS and MRI) [30]. Both the MD and LFD resulted in comparable reductions in body weight. Compared to standard care, MD interventions that focus on specific components, such as the inclusion of additional olive oil (at a dose of 20 grammars or comprising 20% of total fat intake per day), resulted in an increased intake of monounsaturated fatty acids (MUFA) and omega-3, as well as a decrease in the consumption of polyunsaturated fatty acids (PUFA) [9,32]. These interventions demonstrated more pronounced improvements in ALT and AST levels, as well as in the degree of liver steatosis evaluated by ultrasound.

### 3.6. Rates of Dropout and Attrition

In five of the studies, the attrition rates ranged from 10% to 20% [9,28,29,30,32]. For the remaining studies, no data on attrition rates were provided. When reported, the factors that contributed to participant dropout included adverse events [31], noncompliance [9,28,30], relocation to another city [28], personal reasons [30], lack of willingness to continue [9], scheduling conflicts, travel, and health issues [9].

### 3.7. Meta-Analysis and Meta-Regression of the Effects of Olive Oil on NAFLD

Seven trials were included in the meta-analysis [9,27,28,29,30,31,32].

#### 3.7.1. Primary Outcomes

##### Impact of Olive Oil on ALT

**Figure 3:**
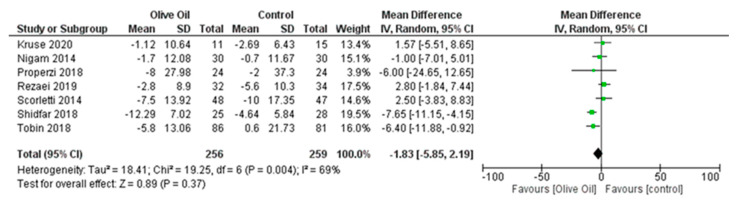
Forest plot of the change in the value (IU/L) of alanine aminotransferase (ALT) using the random-effects model [9,27,28,29,30,31,32].

**Figure 4:**
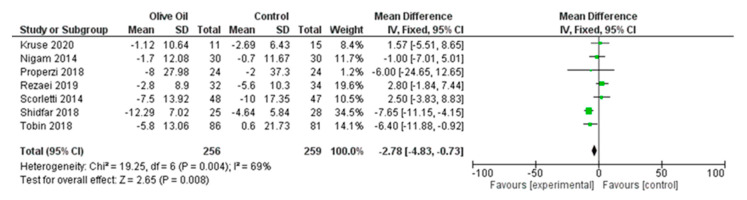
Forest plot of the change in the value (IU/L) of alanine aminotransferase (ALT) using the fixed-effects model [9,27,28,29,30,31,32].

The meta-analysis revealed that olive oil consumption had a positive impact on post-intervention ALT concentrations, although the effect was not statistically significant. However, there was substantial heterogeneity among the trials included in the analysis. On average, the intake of olive oil led to a pooled mean reduction of 1.83 in ALT concentration (ALT IU/L: MD = −1.83, 95% CI: −5.85–2.19, *p* = 0.37, *I*^2^ = 69%) (Figure 3) compared to the control diet. When applying the fixed-effects model, a statistically significant decrease in ALT concentration was reported (Figure 4). However, considering the number of studies included and the overall heterogeneity that exceeds 50%, the most suitable approach to present the findings remains the random-effects model.

#### 3.7.2. Secondary Outcomes

##### Impact of Olive Oil on Other NAFLD Surrogate Markers

**Figure 5:**
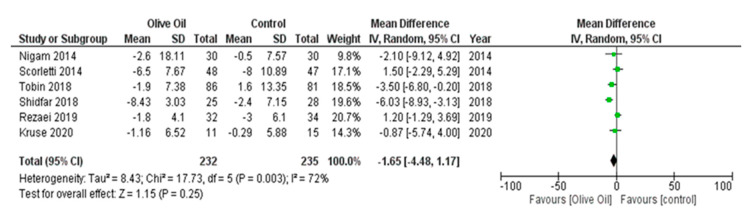
Forest plot of the change in the value (IU/L) of aspartate aminotransferase (AST) using the random-effects model [9,27,28,29,31,32].

**Figure 6:**
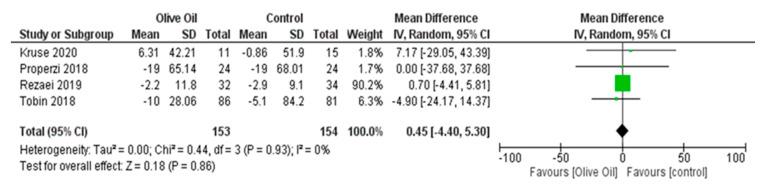
Forest plot of the change in the value (IU/L) of gamma-glutamyl transferase (gGT) using the random-effects model [9,27,30,31].

**Figure 7:**
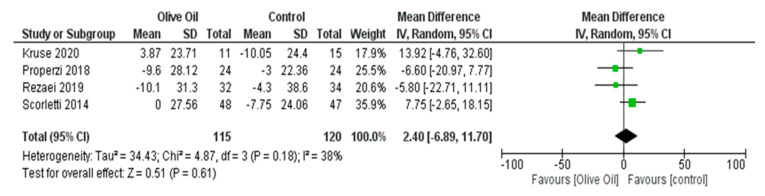
Forest plot of the change in the value (mg/dL) of total cholesterol (TC) using the random-effects model [9,27,29,30].

**Figure 8:**
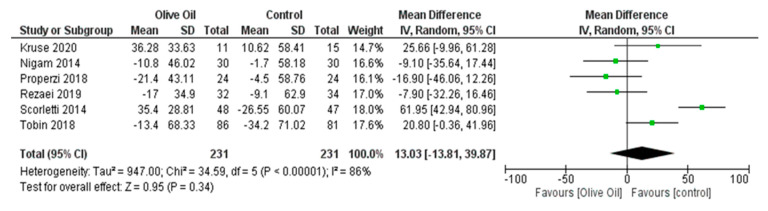
Forest plot of the change in triglyceride value (mg/dL) (TG) using the random-effects model [9,27,28,29,30,31].

**Figure 9:**
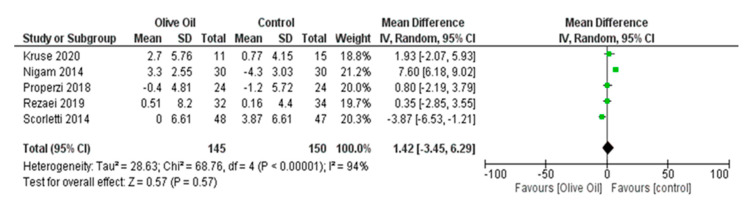
Forest plot of the change in the value (mg/dL) of high-density lipoprotein (HDL-c) using the random-effects model [9,27,28,29,30].

**Figure 10:**
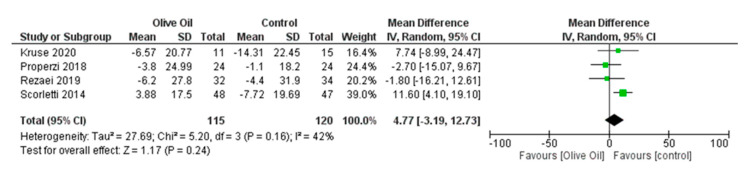
Forest plot of the change in the value (mg/dL) of low-density lipoprotein (LDL-c) using the random-effects model [9,27,29,30].

The intake of olive oil led to a nonsignificant decrease in AST levels (AST IU/L: MD = −1.65, 95% CI: −4.48 to 1.17, *p* = 0.25, *I*^2^ = 72%) (Figure 5) as examined in six out of seven studies. Additionally, olive oil did not have a significant effect on gGT values (gGT IU/L: MD = 0.45, 95% CI: −4.40 to 5.30, *p* = 0.86, *I*^2^ = 0% (Figure 6) or on the lipidemic profile, including TC (TC mg/dL: MD = 2.40, 95% CI: −6.89 to 11.7, *p* = 0.61, *I*^2^ = 38%), TG (TG mg/dL: MD = 13.03, 95% CI: −13.81 to 39.87, *p* = 0.34, *I*^2^ = 86%), HDL-C (HDL-C mg/dL: MD = 1.42, 95% CI: −3.45 to 6.29, *p* = 0.57, *I*^2^ = 94%), and LDL-C (LDL-C mg/dL: MD = 4.77, 95% CI: −3.19 to 12.73, *p* = 0.24, *I*^2^ = 42%) (Figure 7, Figure 8, Figure 9 and Figure 10, respectively).

##### Effect of Olive Oil on Anthropometric Measures

**Figure 11:**
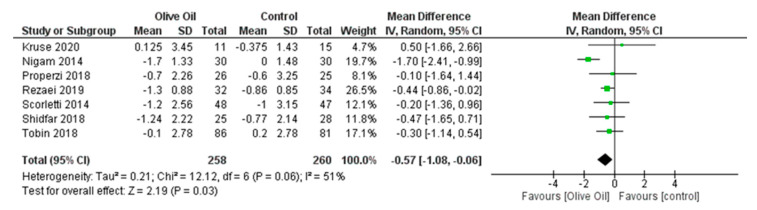
Forest plot of the change in the value (kg/m^2^) of body mass index (BMI) using the random-effects model [9,27,28,29,30,31,32].

**Figure 12:**
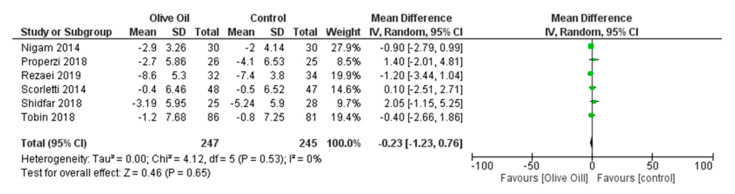
Forest plot of the change in the value (cm) of waist circumference (WC) using the random-effects model [27,28,29,30,31,32].

The meta-analysis showed that olive oil had favorable results on body weight. Taking into account all included studies, a statistically significant reduction in BMI (BMI kg/m^2^: MD = −0.57, 95% CI: −1.08 to −0.06, *p* = 0.03, *I*^2^ = 51%) was observed (Figure 11). Although there was no statistically significant change in WC, there was a noticeable downward trend, as evidenced in three of the six studies (WC cm: MD = −0.23, 95% CI: −1.23 to 0.76, *p* = 0.65, *I*^2^ = 0%) (Figure 12).

**Figure 13:**
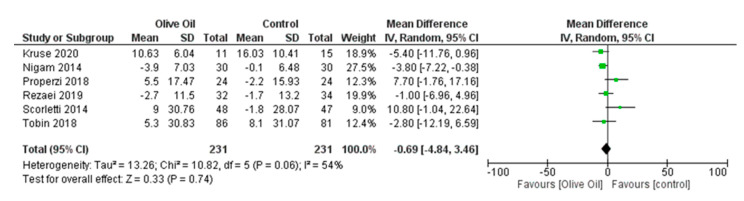
Forest plot of the change in the value (mg/dL) of free plasma glucose (FPG) using the random-effects model [9,27,28,29,30,31].

**Figure 14:**
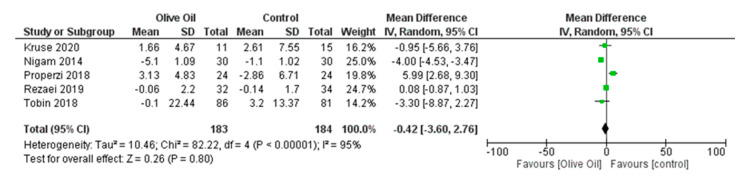
Forest plot of the change in free insulin’s value (μU/mL) (FIns) using the random-effects model [9,27,28,30,31].

**Figure 15:**
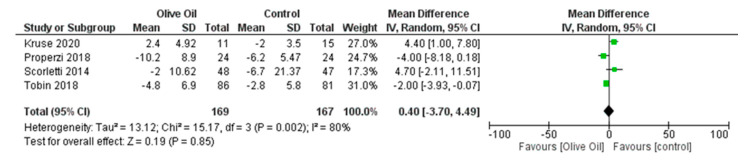
Forest plot of the change in the value (%) of liver fat (LF) using the random-effects model [9,29,30,31].

No significant findings were identified with respect to FPG, FPI levels, and percentage of liver fat. Specifically, for FPG and FPI, the estimated changes were MD = −0.69, 95% CI: −4.84 to 3.46, *p* = 0.74, *I*^2^ = 54%, and MD = −0.42, 95% CI: −3.60 to 2.76, *p* = 0.80, *I*^2^ = 95%, respectively (Figure 13 and Figure 14), while for liver fat percentage, the estimated change was MD = 0.40, 95% CI: −3.70 to 4.49, *p* = 0.85, and *I*^2^ = 80% (Figure 15).

### 3.8. Sensitivity Analysis

The pooled mean difference in the primary outcome did not show statistically significant differences after the sensitivity analysis. However, a noteworthy discovery emerged when the study conducted by Shidfar et al. was omitted from the analysis. This exclusion led to a reduction in initial heterogeneity from 69% to 37% for the ALT concentration (ALT IU/L: MD = −0.33, 95% CI: −3.68 to 3.01, *p* = 0.85, *I*^2^ = 37%) and from 72% to 35% for the AST concentration (AST IU/L: MD = −0.45, 95% CI: −2.59 to 1.69, *p* = 0.68, *I*^2^ = 35%) [32]. Regarding the (BMI), the exclusion of the study by Nigam et al. completely eliminated the existing heterogeneity, reducing it from 51% to 0% while retaining ultimate statistical significance (BMI kg/m^2^: MD = −0.36, 95% CI: −0.69 to −0.04, *p* = 0.03, *I*^2^ = 0%) [28].

Furthermore, when examining the triglyceride concentration, the heterogeneity of the overall effect decreased from 86% to 48% (TG mg/dL: MD = 2.19, 95% CI: −14.31 to 18.70, *p* = 0.79, *I*^2^ = 48%) following the removal of the Scorletti et al. study [29], without statistically significant impact on the outcome. Lastly, the risk of bias associated with individual studies was evaluated. Excluding studies that demonstrated an elevated overall risk of bias (including some concerns and high risk) resulted in reduced heterogeneity, which did not produce significant deviations from the study outcomes.

Using the fixed-effects model for the ALT, AST, HDL, LDL, and Fins outcomes led to statistically significant results (Figure 2). With subsequent sensitivity analysis leading to *I*^2^ < 50%, the random- and fixed-effects models yielded the same results. Consequently, the random-effects model was used given the substantial heterogeneity exhibited among the included studies.

## 4. Discussion

The present study entails an updated and systematic evaluation of the current literature on the possible beneficial role of olive oil consumption in NAFLD. Seven studies with 529 individuals were integrated, focusing on changes in liver function markers and body weight after dietary interventions. Although olive oil consumption or supplementation did not produce a statistically significant improvement in liver biochemistry, it led to a significant decrease in mean BMI levels of subjects with NAFLD. To the best of our knowledge, this is the first meta-analysis on the effects of olive oil on NAFLD. Numerous systematic reviews and meta-analyses have demonstrated the favorable effects of the MD on NAFLD, positively influencing a variety of variables such as ALT concentrations, liver fat percentage, and HOMA-IR scores [38,39,40]. Unfortunately, a recently published systematic review that addresses the impact of olive oil on liver steatosis and enzyme levels did not include a meta-analysis [41].

Olive oil, a critical component of MD, is often used synonymously with this nutritional advocacy [42]. Consumption of olive oil, which serves as the main source of fat in MD, has been associated with numerous health benefits mediated by its antioxidant and anti-inflammatory properties. Uli et al. demonstrated that the MD has the potential to effectively reduce LDL, TG, TC, and FBG levels while simultaneously increasing HDL-C concentrations in people with overweight and obesity [43]. A recent meta-analysis of 13 RCTs, investigating the comparative efficacy of the MD versus the conventional LFD on various metabolic outcomes among subjects at high cardiovascular risk who reside in non-Mediterranean regions, revealed that an MD improved TC levels. However, no statistically significant effects were observed for the other lipid profile markers [44]. People with a high adherence to an MD exhibit significant reductions in BMI and body weight [45]. A systematic review and meta-analysis focusing on adherence to an MD revealed a notable decrease in WC [46]. Furthermore, other meta-analyses have also reported favorable effects of an MD on body weight, WC, and other anthropometric parameters in subjects diagnosed with NAFLD [47,48]. It is plausible that our meta-analysis yielded different results due to the characteristics of the primary studies involved. Small sample sizes may have limited these studies, thereby influencing the overall findings. Furthermore, our focus specifically on olive oil, rather than on the broader MD, could have contributed to the variations in results. This highlights the importance of considering the specific context and scope of the primary studies when interpreting the findings of a meta-analysis.

While olive oil offers various health benefits overall, it is important to acknowledge that the quality and storage methods employed can diminish its health effectiveness. Additionally, the choice of olive oil and awareness of its quality play crucial roles. Many consumers opt for olive oil regardless of brand yet not all opt for EVOO, which holds superior nutritional value. A study conducted by Marakis et al. in Greece revealed that a significant portion of participating households (60.3%) utilized olive oil produced by themselves, their extended family, or friends, while only 27.4% purchased branded olive oil. Moreover, approximately 57% reported using EVOO [49].

As is widely recognized, olive oil is a vegetable oil rich in monounsaturated fatty acids, most notably oleic acid, while maintaining low levels of saturated fatty acids. This oil boasts a range of potent antioxidant polyphenols, vitamin E, vitamin K, and various other natural antioxidants. Among these polyphenols, oleuropein, oleocanthal, and hydroxytyrosol emerge as the most significant contributors, conferring nutritional and anti-inflammatory benefits. Mechanisms driving the optimal effects of olive oil on body weight, glycemia, and insulin sensitivity involve the regulation of glucose production and uptake, insulin signaling pathways in the liver and white adipose tissue, and PPARγ/α activation by oleanolic acid [50]. Recently, a study carried out in mice investigating the effects of various vegetable oils on NAFLD revealed that canola oil and olive oil not only induced reductions in body weight but also led to a decrease in liver and retroperitoneal fat, ultimately resulting in favorable changes in liver histology [51]. Patti et al. uncovered significant benefits associated with consuming EVOO rich in oleocanthal over a two-month period. Their study revealed marked reductions in body weight, BMI, and waist circumference despite no accompanying lifestyle or dietary habit alterations. These favorable outcomes are believed to stem from heightened postprandial fat oxidation, a phenomenon commonly observed following the consumption of meals containing olive oil [52]. Moreover, the adoption of an MD enriched with EVOO emerges as a promising alternative to low-fat diets for weight maintenance among older adults grappling with overweight or obesity. In line with these findings, a meta-analysis conducted in 2020 further underscored the efficacy of olive oil in reducing BMI, demonstrating statistically significant reductions (*p* < 0.001). Notably, the benefits were most pronounced when olive oil was consumed in its natural liquid form, particularly after cooking, as opposed to when administered in capsule form [53].

Although the present study did not directly establish substantial changes in liver enzymes and the percentage of liver fat due to olive oil supplementation, it provided evidence of an indirect influence on the progression of NAFLD. Considering that all major scientific societies advocate weight reduction and lifestyle adjustments as fundamental components of treatment for patients with NAFLD [54,55,56,57], it is important that olive oil can help people with this condition improve their anthropometric profile. 

Notably, olive oil intake, even in the absence of calorie restriction, has been shown to exhibit beneficial effects on NAFLD, even with minimal weight loss (approximately 2%) [30]. However, specific studies have reported that calorie-restricted MD interventions can lead to substantial weight loss, ranging from 5% to 10%, achieving the goals suggested by the European Association for the Study of Liver Clinical Practice Guidelines [8,58,59,60,61]. Given that the magnitude of weight loss promoted by olive oil itself is relatively small, the combination of calorie restriction and olive oil consumption emerges as a promising strategy to induce the recommended degree of weight reduction and to promote notable improvements in NAFLD. Furthermore, the protective effects of olive oil and an MD on the metabolic disturbances associated with MetS, which are strongly correlated with NAFLD, have been well documented [22,38,48]. Finally, the low dropout rates observed in studies involving olive oil-based interventions suggest that olive oil serves as a well-tolerated dietary intervention for this specific population.

### Strengths and Limitations and Areas for Future Research

The primary objective in evaluating diet lifestyle interventions is twofold: first, to determine their feasibility and practicality if proven effective, and second, to identify specific individuals or subgroups that can derive significant benefits from a particular intervention. However, in most studies, the assessment of dietary intake was typically based on self-report instruments, each of which is associated with well-documented risks of misreporting, errors, and bias. Although comparing a specific diet approach with a constant placebo or standard diet is challenging, these limitations are ubiquitous in most dietary intervention trials. The dosage of olive oil administered varied widely, ranging from 3 g/d to ad libitum. As a result, it is imperative for future studies to determine the minimum effective dose of the intervention. Additionally, considering the diverse age ranges of the subjects in the studies, it is crucial to assess whether female participants are still of childbearing age or have already reached menopause. This distinction is vital for accurately understanding the intervention’s potential impact across different reproductive health stages in women.

Furthermore, approximately half of the trials used MRI as a diagnostic tool, which is the most accurate method among imaging surrogates to assess HS, while the other half resorted to ultrasound, a more practical tool, although with certain limitations. In the case of LSM, only one trial reported its usage. Although liver biopsy remains the current gold standard to assess the severity of NAFLD, it was not performed in any trial. Consequently, the need for precise noninvasive methods for the evaluation of NAFLD remains to be addressed in future studies. 

The primary mode of intervention predominantly involved individual face-to-face consultations, with fewer studies using group sessions and telephone contact. The responsibility for implementing interventions targeting dietary components was primarily in the hands of nutritionists and dietitians. Additionally, it is noteworthy that lifestyle modifications, weight loss maintenance strategies, and physical activity were described in at least half of the studies analyzed. Given this, it is conceivable that the observed decrease in BMI among individuals with NAFLD may be attributed to these multifaceted interventions, which encompassed more than just olive oil consumption. This underscores a limitation inherent in the primary studies themselves. However, specific behavior change strategies that effectively enhance intervention outcomes remain uncertain. Future research must prioritize this aspect to optimize intervention delivery, promote sustained change in diet behavior, and maximize benefits in terms of liver function. Understanding and implementing effective behavior change techniques will be key to ensuring the success of interventions and improving the general health outcomes of people with NAFLD.

## 5. Conclusions

Olive oil continues to be a safe and easily accessible dietary option with potential benefits for individuals with NAFLD. This meta-analysis indicates that the incorporation of olive oil into the diet can lead to modest, but still significant, improvements in anthropometry. Additionally, the unique properties of olive oil, including its prominent role in the MD and its well-established advantages for cardiovascular health, general well-being, and longevity, position it as an excellent recommendation for regular consumption in all age groups [62]. However, there is limited evidence on factors that influence individual patient decisions to adopt and maintain dietary changes for NAFLD management. To improve future studies in this area, it is essential to perform robust evaluations of dietary intake and the acceptability and sustainability of the various interventions. Additionally, including measures related to quality of life and patient outcomes would be valuable.

The ideal dietary intervention for patients with NAFLD remains uncertain. A recent study found that a high-fat diet (HFD) can disrupt redox balance, leading to metabolic dysfunction. The study assessed oxidative stress biomarkers and their correlation with MetS using specific assays. After 8 weeks of HFD, MetS-like symptoms and a hepatic profile resembling NAFLD emerged, marked by elevated hydroperoxide levels. These biomarkers could aid in evaluating redox balance, the effectiveness of antioxidant therapies for MetS, and the potential degeneration of NAFLD [63]. Significant heterogeneity among the reviewed studies limits the generalizability of the findings to clinical practice. Furthermore, most of the trials were short- to medium-term in duration, and their results need to be confirmed through larger, longer-term studies with consistent outcome measures. Addressing these areas of uncertainty and incorporating personalized nutrition intervention approaches can significantly improve the care and outcomes of patients with NAFLD. By better understanding the factors that influence patient dietary choices and long-term adherence, clinicians can tailor interventions to meet individual needs and optimize treatment of this growing health challenge.

## Figures and Tables

**Table 1:** Risk of bias using the Cochrane tool RoB2.

Unique ID	Study ID	Experimental	Comparator	Outcome	W	D1	D2	D3	D4	D5	Overall
Kruse 2020 [27]	DOI: 10.1002/mnfr.202000419	Olive Oil (OL)	Rapeseed Oil (RA)	ALT	1	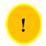	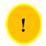	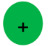	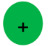	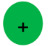	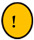
Nigam 2014 [28]	DOI: 10.1089/dia.2013.0178	Olive Oil or Canola	Control Oil	ALT	1	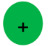	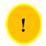	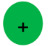	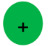	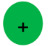	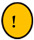
Scorletti 2014 [29]	DOI: 10.1002/hep.27289	Omacor	Olive Oil	ALT	1	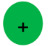	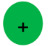	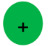	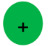	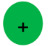	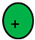
Properzi 2018 [30]	DOI: 10.1002/hep.30076	MED diet	Low-Fat Diet	ALT	1	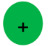	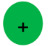	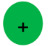	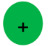	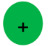	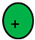
Rezai 2019 [9]	DOI: 10.1016/j.nut.2018.02.021	Olive Oil	Sunflower Oil	ALT	1	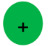	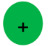	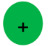	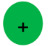	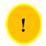	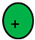
Tobin 2018 [31]	DOI: 10.3390/nu10081126	MF4637	Olive Oil	ALT	1	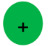	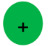	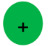	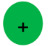	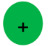	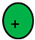
Shidfar 2018 [32]	DOI: 10.1155/2018/1053710	Olive Oil	Control	ALT	1	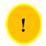	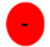	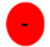	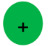	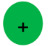	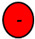
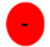 High Risk 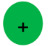 Low Risk 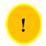 Some Concerns		D1: Randomization process D2: Deviations from the intended interventions D3: Missing outcome data			D4: Measurement of the outcome D5: Selection of the reported result W: Weight						

**Table 2:** Baseline characteristics of the included studies.

Study	Design	Clinical Group	Follow-Up (weeks)	Intervention/Control Groups	Number of Patients, Male/Female (%)	Age—Mean (years)	BMI—Mean (kg/m^2^)	Intervention	Intervention Features
Properzi 2018, Australia [30]	RCT	NAFLD	12	Intervention group **	26, (57.7%)/(42.3%)	51	31.5	MD—based on the traditional Cretan diet (40% CHO, 20% PRO, 35–40% FAT, and <10% SFA).	750 g nuts, 750 mL olive oil (MD), 1 kg of natural muesli, and 200 g of low-fat snack bars every 4 weeks (LFD).
				Control group	25, (44%)/(56%)	53	30.2	Low-fat/high-CHO (LFD)—based on National Health and Medical Research Council and AHA guidelines (50% CHO, 20% PRO, 30% FAT, and <10% SFA).	
Rezaei 2019, Iran [9]	RCT	NAFLD	12	Intervention group **	32, (37.5%)/(62.5%)	46.3	30.6	MD component—increased olive oil intake (20 g/day).	Cal restriction: 500 cal/d deficit (both). Physical activity: Moderate intensity 30–40 min/d (both). Olive oil and sunflower oil dosages are provided (both). 50–55% CHO, 10–15% PRO and 30–35% FAT (both).
				Control group	34, (50%)/(50%)	40.8	29.6	Increased sunflower oil intake (20 g/day).	
Nigam 2014, India [28]	RCT	NAFLD	24	Intervention group **	30, (100%)/0	37.2	27.2	Olive oil (not exceeding 20 g/day).	Cal intake: 15–21% PRO, 55–70 CHO, 20% FAT (both). Physical activity: Moderate intensity 40–45 min/d (both). Therapeutic lifestyle changes were given (both).
				Intervention group	33, (100%)/0	38	27.4	Canola oil (not exceeding 20 g/day).	
				Control group	30, (100%)/0	36.2	27.4	Refined oil (not exceeding 20 g/day).	
Scorletti 2014, United Kingdom [29]	RCT	NAFLD	72	Intervention group	51, (49%)/(51%)	48.6	34.3	Omacor (gel capsules contained 460 mg EPA and 380 mg DHA)—4 g/day	General dietary advice and education information were provided without advice on calorie allowances, physical activity, or behavior changes (both).
				Control group **	52, (67.3%)/(32.7%)	54	32	Olive oil (gel capsules contained 1 g of olive oil)—4 g/day	
Kruse 2020, Germany [27]	RCT	NAFLD	8	Intervention group **	11, (100%)/0	54	33.1	Increased olive oil intake (50 g/day).	Modification of energy intake according to the weight (>1 kg from the initial body weight). No advice given on physical activity or behavior changes (both).
				Control group	16, (100%)/0	58	32	Increased rapeseed oil intake (50 g/day).	
Tobin 2018, United States [31]	RCT	NAFLD	24	Intervention group	87, (44.4%)/(55.6%)	55.3	32.1	Omega-3 MF46367 (gel capsules containing 460 mg EPA and 380 mg DHA)—3 caps daily.	Cal restriction: Reduced regular caloric intake. Consumption of 2 meals of omega-3-rich fish per week and reduction in consumption of foods rich in trans and omega-6 fatty acids. Physical activity: Stable levels.
				Control group **	89, (51.1%)/(48.9%)	55.1	32.4	Olive oil (gel capsules containing 1 g of olive oil)—3 caps daily.	
Shidfar 2018, Iran [32]	RCT	NAFLD	12	Intervention group **	25, (61.9%)/(38.1%) *	46.14	29.64	MD component—increased olive oil intake (20% of total fat).	Cal restriction: Personalized Cal deficit (both). Olive oil dosage supplied. 50% CHO, 20% PRO and 30% FAT (both).
				Control group	25, (59.1%)/(40.9%) *	45.68	29.9	Healthy diet lifestyle (sunflower oil).	

MD: Mediterranean diet, RCT: randomized controlled trial, NAFLD: nonalcoholic fatty liver disease, Cal: calorie, CHO: carbohydrate, PRO: protein, LFD: low-fat diet, AHA: American Heart Association, EPA: eicosapentaenoic acid, DHA: docosahexaenoic acid. *: percentage available after dropout, **: the group encompassed olive oil.

**Table 3:** Impact of dietary interventions (olive oil) on primary and secondary outcomes.

Study	Outcome Measures in Study	Attrition/Dropout	Primary Outcomes	Secondary Outcomes
Properzi 2018, Australia [30]	HS (MRS-PDFF), LSM [TE FibroScan™ (Echocens, Paris, France)], HepaScore, ALT, cardiometabolics, anthropometry, and QoL	Intervention: 2/26, 7.7% Control: 1/25, 4.0%	↓ HS and ↓ ALT (both) HS RR: −32.4% ± 25.5% vs. −255.0% ± 25.3% (intervention vs. control)	↓ gGT, HbA1c, TC, TG and FRS (intervention) ↓ WC and ↑ QoL (both) ↓ BW (both) BW: −2.3% vs. −2.1% (intervention vs. control)
Rezaei 2019, Iran [9]	HS (US), ALT, AST, cardiometabolics, anthropometry and diet record diaries	Intervention: 6/32, 18.8% Control: 6/34, 17.6%	↓ HS (both) ↓ ALT (control) ↓ HS (intervention vs. control)	↓ AST (both) ↓ TG (intervention) ↓ BW, WC, and systolic/diastolic BP (both) BW: −4.1% vs. −2.9% (intervention vs. control)
Nigam 2014, India [28]	LF (US), AST, ALT, anthropometry, cardiometabolics, and food questionnaire	Intervention: 0/30, 0% Intervention: 0/30, 0% Control: 3/33, 9.1%	↓ LF (intervention) ↓ ALT (intervention)	↓ AST, TC, TG (intervention) ↑ HDL-C (intervention) ↓ FBG (intervention vs. control) ↓ FBG (olive oil vs. canola oil) ↓ BW (olive vs. control)
Scorletti 2014, United Kingdom [29]	HS (MRS), diet questionnaire, ALT, AST, cardiometabolics, and anthropometry	Intervention: 4/51, 7.8% Control: 4/52, 7.7%	↓ ALT (control)	↓ HS and TG (intervention) ↓ AST (control) ↓ HDL-C (intervention)
Kruse 2020, Germany [27]	HS (H-MRS), ALT, AST, anthropometry, cardiometabolics, and diet record diaries	Intervention: 0/11, 0% Control: 1/16, 6.3%	↓ ALT (both) ↑ IHL (intervention vs. control)	↓ LDL, AST (intervention) ↑ HDL (intervention) No changes BW, TG (both)
Tobin 2018, United States [31]	LF (MRI-PDFF), ALT, AST, anthropometry, and cardiometabolics	Intervention: 6/87, 6.9% Control: 3/89, 3.4%	↓ ALT (control)	↓AST and gGT (control) ↓ WC (control) ↓ in LF percentage (both) ↓ TG 18% vs. 7% (intervention vs. control)
Shidfar 2018, Iran [32]	HS (US), ALT, AST, anthropometry, and diet questionnaire	Intervention: 4/25, 16.0% Control: 3/25, 12.0%	↓ ALT (both) ↓ ALT and HS (intervention vs. control)	↓ AST (intervention) ↓ WC (both) ↓ BW (both) BW: −4.3% vs.−3.5% (intervention vs. control)

AST: aspartate aminotransferase, ALT: alanine transaminase, BW: body weight, WC: waist circumference, HS: hepatic steatosis, US: ultrasound, MRS: magnetic resonance spectroscopy, H-MRS: proton magnetic resonance spectroscopy, MRS-PDFF: magnetic resonance spectroscopy–measured proton density fat fraction, MRI-PDFF: magnetic resonance imaging–measured proton density fat fraction, IHL: intrahepatic lipid content, HDL-C: high-density lipoprotein cholesterol, gGT: gamma-glutamyl transferase, FBG: fasting blood glucose, TG: triglyceride, TC: total cholesterol, LDL: low-density lipoprotein, LF: liver fat, BP: blood pressure, LSM: liver stiffness measurement, FRS: Framingham risk score, QoL: quality of life, TE: transient elastography, ↓ Decreased, ↑ Increased.
